# HER2/neu expression correlates with vasculogenic mimicry in invasive breast carcinoma

**DOI:** 10.1111/j.1582-4934.2012.01653.x

**Published:** 2012-12-19

**Authors:** Tieju Liu, Baocun Sun, Xiulan Zhao, Qiang Gu, Xueyi Dong, Zhi Yao, Nan Zhao, Jiadong Chi, Ning Liu, Ran Sun, Yuemei Ma

**Affiliations:** a Department of Pathology, Tianjin Medical UniversityTianjin, China; b Department of Pathology, Tianjin Cancer Hospital, Tianjin Medical UniversityTianjin, China; c Department of Pathology, Tianjin General Hospital, Tianjin Medical UniversityTianjin, China

**Keywords:** HER2, vasculogenic mimicry, breast cancer

## Abstract

Vasculogenic mimicry (VM) refers to the condition in which tumour cells mimic endothelial cells to form extracellular matrix-rich tubular channels. VM is more extensive in more aggressive tumours. The human epidermal growth factor receptor 2 (HER2) gene is amplified in 20–30% of human breast cancers and has been implicated in mediating aggressive tumour growth and metastasis. However, thus far, there have been no data on the role of HER2 in VM formation. Immunohistochemical and histochemical double-staining methods were performed to display VM in breast cancer specimens. Transfection in MCF7 cells was performed and clones were selected by G418. The three-dimensional Matrigel culture was used to evaluate VM formation in the breast cancer cell line. According to statistical analysis, VM was related to the presence of a positive nodal status and advanced clinical stage. The positive rate of VM increased with increased HER2 expression. In addition, cases with HER2 3+ expression showed significantly greater VM channel count than those in other cases. The exogenous HER2 overexpression in MCF-7 cells induced vessel-like VM structures on the Matrigel and increased the VM mediator vascular endothelial (VE) cadherin. Our data provide evidence for a clinically relevant association between HER2 and VM in human invasive breast cancer. HER2 overexpression possibly induces VM through the up-regulation of VE cadherin. Understanding the key molecular events may provide therapeutic intervention strategies for HER2+ breast cancer.

## Introduction

The human epidermal growth factor receptor 2 (HER2/neu) gene is identified as a proto-oncogene that is amplified and/or overexpressed in 20–30% of human breast cancers [1]. Many aspects of tumour growth are favourably affected through the activation of HER2 signalling [2, 3]. Currently, a number of breast cancer therapies inhibit the HER2 signalling pathway [4–8]. Although the biological pathways activated by HER2 are not yet completely characterized, the oncogenic potential of HER2 has been clearly established. Breast cancer cells transfected with HER2 acquire a more malignant phenotype, with increased cell invasion, angiogenesis and metastasis [9].

The growth and metastasis of tumours has been thought to be an angiogenesis-dependent process. However, a non-angiogenesis-dependent pathway in which tumours can feed themselves has also been reported. The condition in which tumours feed themselves using alternative pathways without the participation of endothelial cells is known as vasculogenic mimicry (VM) [10–13]. VM patterns are present in numerous malignant tumour types, including mesenchymal tumours [12, 14]. This condition has also been reported recently in epithelial carcinoma, such as hepatocellular carcinoma [11], invasive breast carcinoma [10] and ovarian carcinoma [15, 16]. VM represents the formation of perfusion pathways by tumour cells. The physical connection between VM and blood vessels may also facilitate haematogeneous dissemination of tumour cells. Therefore, the presence of VM in tumours is related to a more aggressive biological behaviour. Interestingly, a previous study showed that the vascular endothelial growth factor expression is positively correlated with HER2 expression in human breast carcinomas [17–20]. However, thus far, no data on the role of HER2 in VM formation have been reported.

## Materials and methods

### Tissue specimens

The tissue collection and analysis conducted in this study was approved by the Ethical Committee of Tianjin Medical University, China. All breast surgical specimens were formalin fixed and paraffin embedded by the Pathology Department, Tianjin Medical University. All cases were randomly selected from 2004 to 2007 and were made anonymous to us. The pathologic diagnosis was counterchecked by two senior pathologists according to the 2003 World Health Organization histological classification of breast tumours.

### CD31/periodic acid-Schiff (PAS) immunohistochemical and histochemical double-staining methods

Tissue sections (5 μm) were deparaffinized and hydrated utilizing standard procedures. Endogenous peroxidase activity was blocked with 3% hydrogen peroxide in 50% methanol for 30 min. at room temperature. The sections were rehydrated and washed with phosphate-buffered saline (PBS). Antigen retrieval was conducted by treating the tissue sections with citrate buffer (0.01 M citric acid, pH 6.0) for 20 min. at 95°C in a microwave oven. Non-specific binding sites were blocked by exposure to 10% normal goat serum in PBS for 30 min. at 37°C. The sections were then incubated overnight at 4°C with anti-CD31 antibody (Cat.# TA500124; Beijing Zhongshan Biotechnology Limited Company, Beijing, China) at a dilution ratio of 1:40. PBS was used as the negative control in place of primary antibodies. The sections were rinsed with PBS and incubated with biotinylated goat antimouse immunoglobulin G for 30 min. at 37°C. Then, the sections were incubated with 3,3′-diaminobenzidine chromogen for 10 min. at room temperature and washed with distilled water.

Finally, the sections were treated with PAS staining. All sections were oxidized in 0.5% periodic acid solution for 5 min., rinsed in distilled water, placed in Schiff reagent for 15 min. and washed in tap water for 5 min. Then, the sections were counterstained with haematoxylin, dehydrated in graded ethanol solutions (75–100%), cleared with xylene and mounted with neutrogum.

### VM identification by CD31/PAS colabelling

**Fig. 1:**
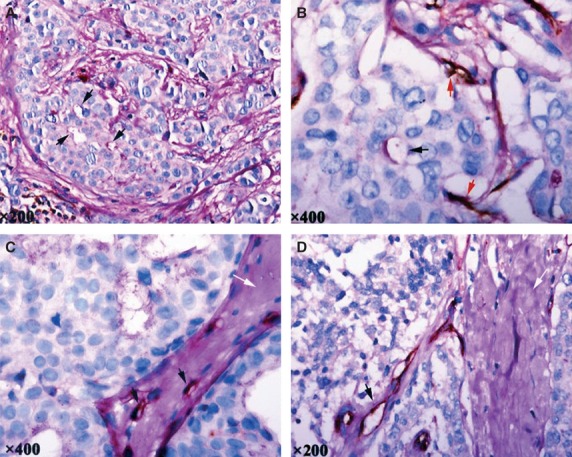
The identification of VM in invasive breast cancer specimens. (**A**) and (**B**) CD31-negative, PAS-positive vascular-like channels with or without red blood cells formed by breast cancer cells were counted as positive for VM (black arrow). Typical blood vessels were CD31-positive (red arrow). (**C**) and (**D**) Breast cancer without VM showed that a PAS-positive matrix is mainly present in the mesenchyme of the breast cancer tissue (white arrow) and has a close relationship with blood vessels (black arrow).

CD31 and PAS immunohistochemical and histochemical double staining was used to identify VM. CD31-negative, PAS-positive vascular-like patterns containing red blood cells formed by breast cancer cells were identified as positive for VM [10, 11] (Fig. 1A–B, black arrow). VM channels were not lined by endothelial cells, as proven by the lack of CD31 (brown) staining. In addition, the cells forming VM showed the morphology of the surrounding breast cancer cells (Fig. 1B). The breast cancer cells mimicked the endothelial cells to form extracellular matrix-rich channels (PAS-positive). Necrotic and inflammatory cells infiltrating around the channels were absent. By contrast, typical blood vessels showed positive co-labelling of CD31 and PAS (Fig. 1B, red arrow; C–D, black arrow). Positive staining by CD31 and PAS was observed on the luminal surface and in the wall of the blood vessels, respectively.

Breast cancer without VM showed a PAS-positive matrix mainly present in the stroma (Fig. 1C–D, white arrow). The PAS-positive material had a close relationship with the blood vessels (Fig. 1C–D, black arrow).

### VM channel quantification

Vasculogenic mimicry quantification was assessed by light microscopic analysis in tumour areas that contained the most VM channels (so-called ‘hot spots’). The hot spot was identified by scanning at low power (×100 and ×200). Ten non-overlapping fields at ×400 magnification were chosen to determine the median value of the VM channels. The number of VM channels within the hot spot was assessed using an ocular grid and the forbidden lines method to facilitate the counting.

### Cell culture and transfection

The breast cancer cell line used in this study was MCF-7 from the American Type Culture Collection, Rockville, MD, USA. Cells were cultured in Dulbecco's modified Eagle's medium supplemented with 10% foetal bovine serum (FBS). Transfection of MCF7 cells was done using lipofectamine 2000, and clones were selected with G418. Single clones were analysed for their content of transfected proteins by Western blot with an anti-HER2 antibody (1:500, Cat.#MS-730-P0; Thermo SCIENTIFIC, Rockford, IL, USA).

### Migration assays

Transwell migration assays were conducted on MCF7 cells and MCF7 cells with HER2 overexpression (MCF-7^HER2^). Transwell chambers (8 μM pore) were first coated with 10 μg/mL fibronectin overnight at 4°C, washed in PBS and rehydrated with serum-free media for 30 min. at 37°C. The media were removed, and then the cells (1 × 10^5^/chamber) were plated in the upper chambers of the serum-free media. The lower chambers contained media with 15% FBS as a chemo attractant. Chambers were incubated for 3 hrs at 37°C and 5% CO_2_. Migrated cells were stained with crystal violet solution and counted in a ×200 field.

### Western blot analysis

The whole cell lysates were resolved by sodium dodecyl sulphate-polyacrylamide gel electrophoresis and transferred onto polyvinylidene difluoride membranes. Blots were blocked and incubated with the monoclonal antibody HER2 (1:500, Cat.#MS-730-P0; Thermo SCIENTIFIC, Rockford, IL, USA) and VE-cadherin (1:500, Cat.#ab33168; Abcam, Cambridge, UK), followed by incubation with a secondary antibody (1:2000, Cat.#sc-2055, Cat.#sc-2004, Santa Cruz, CA, USA). Blots were developed using an enhanced chemiluminescence detection kit (Amersham Pharmacia Biotech, Piscataway, NJ, USA). For protein loading analyses, a monoclonal β-actin antibody (1:2000, Cat.#sc-47778; Santa Cruz, CA, USA) was used.

### Three-dimensional (3D) cultures

The tumour cell mixtures were seeded with Matrigel to allow them to polymerize. The addition of regular medium was performed during the incubation. Cells were photographed using a phase contrast microscope.

### Statistical analysis

The data analysis was performed using the SPSS 16.0 software package. All *P* values were two sided, and the statistical significance was set at *P* = 0.05.

## Results

### Relationship of VM and clinicopathological data in invasive breast cancer

Twenty-seven (22.5%) cases with VM were identified out of the 120 cases of invasive breast cancer specimens. The clinicopathological data in patients with VM (*n* = 27) were compared with those without VM formation (*n* = 93) in breast cancer (Table 1).

**Table 1:** The relationship of VM and clinicopathological data in invasive breast cancer

Clinicopathological parameters	VM formation		VM positive rate (%)	*P* value
	Positive (*n* = 27)	Negative (*n* = 93)		
Age (years)	47.30 ± 0.62	47.67 ± 0.25		0.600
Nodal status				0.027
Positive	16	33	32.7	
Negative	11	60	15.5	
Differentiation grade				0.456
G1	10	23	30.3	
G2	7	31	18.4	
G3	10	39	20.4	
Histological type,				0.083
Ductal NOS	19	80	19.2	
Lobular	8	13	38.1	
Tumour size (mm)	24.78 ± 1.03	25.06 ± 0.48		0.828
Tumour stage (UICC)				0.022
I	7	52	11.9	
II	16	34	32.0	
III	4	7	36.4	
ERα or PR status				0.143
Positive	20	80	20.0	
Negative	7	13	35.0	
HER2 status				0.018
0/+	12	69	14.8	
++	8	13	38.1	
+++	7	11	38.9	

Among all of the factors compared, the presence of nodal status and clinical stage were significantly different between groups with VM and without VM (*P* < 0.05). VM was observed in 16 node-positive cases (32.7%) and 11 node-negative cases (15.5%), and this difference was significant (*x*^*2*^ = 4.896, *P* = 0.027). VM was also present in 7 cases (11.9%) with stage I, 16 cases (32.0%) with stage II and 4 cases (36.4%) with stage III breast cancer (*x*^*2*^ = 7.628, *P* = 0.022). The positive rate of VM was significantly higher in the progressive stage (II and III) than in the primary stage (I) of breast cancer (32.8% *versus* 11.9%) (*x*^*2*^ = 7.529, *P* = 0.006). Therefore, VM was positively associated with the poor outcome in patients. No significant differences between the groups with VM and without VM with respect to age (*t* = 0.526, *P* = 0.600), tumour size (*t* = 0.217, *P* = 0.828), differentiation grade (*x*^*2*^ = 1.638, *P* = 0. 456) and histological type (*x*^*2*^ = 3.550, *P* = 0.083) were found.

### More VM was present in breast cancers with increased HER2 expression

**Fig. 2:**
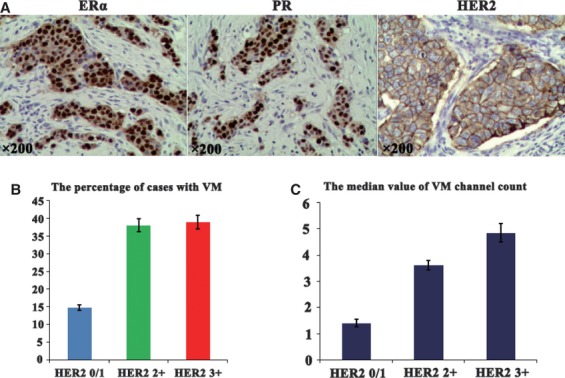
More VM was formed in breast cancer with increased HER2 expression. (**A**) ERα-positive, PR-positive and HER2-positive expression in invasive breast cancer. (**B**) The positive rate of VM showed a sharp increase in HER2 2+ and HER2 3+ compared with HER2 0/1+ expression. (**C**) VM channel counting was performed in cases with VM formation. The median value of the VM channel count in HER2 3+ was significantly greater than either the HER2 2+ or HER2 0/1+ expression.

The assessment of oestrogen receptor (ERα), progesterone receptor (PR) and HER2 is routinely performed in every breast cancer patient for prognosis and to select candidates for hormonal and anti-HER2 therapy (Fig. 2A).

In this study, 100 (83.3%) patients were ERα or PR positive, whereas 20 (16.7%) patients were receptor negative (Table 1). The presence of VM did not demonstrate any correlation with receptor status (*x*^*2*^ = 2.151, *P* = 0.143).

Human epidermal growth factor receptor 2 expression was rated 0/1+ in 81 (67.5%) patients, 2+ in 21 (17.5%) patients and 3+ in 18 (15.0%) patients (Table 1). Our results show that VM was present in 12 cases (14.8%) with HER2 0/1+ expression, 8 cases (38.1%) with HER2 2+ expression and 7 cases (38.9%) with HER2 3+ expression (Fig. 2B). The positive rate of VM showed a sharp significant increase with increased HER2 expression (*x*^*2*^ = 8.036, *P* = 0.018). Both HER2 2+ and HER2 3+ showed greater VM-positive rates than HER2 0/1+ (*x*^*2*^ = 4.352, *P* = 0.037 and *x*^*2*^ = 4.061, *P* = 0.044, respectively). Interestingly, the positive rate of VM was elevated to a similar extent in HER2 2+ and HER2 3+ (*x*^*2*^ = 0.003, *P* = 0.959). The combination of cases with HER2 2+ and HER2 3+ showed greater VM-positive rate than HER2 0/1+ (*x*^*2*^ = 8.442, *P* = 0.004).

Vasculogenic mimicry channel counting was then performed in cases with VM formation. VM was observed in 12 HER2 0/1+ cases, 8 HER2 2+ cases and 7 HER2 3+ cases. The median value of the VM channel count was 1.42 ± 0.15 in HER2 0/1+, 3.63 ± 0.18 in HER2 2+ and 4.86 ± 0.34 in HER2 3+ (Fig. 2C). Significant differences were observed among the three groups with different HER2 expressions (*F* = 71.280, *P* = 0.000). The highest median value of the VM channel count was present in HER2 3+ cases. Furthermore, a significant difference in the VM numbers was observed when HER2 3+ was compared with either HER2 2+ or HER2 0/1+ (*t* = 3.308, *P* = 0.006 and *t* = 10.698, *P* = 0.000, respectively). Therefore, the presence of VM was closely associated with the increased HER2 expression in breast cancer.

### Effects of HER2 overexpression on *in vitro* VM formation in breast cancer cells

**Fig. 3:**
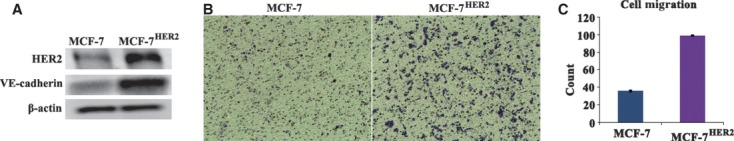
Constitutive activation of HER2 induced the increased cell migration and VE-cadherin expression. (**A**) MCF-7 transfected with HER2 cDNA expressed higher levels of HER2 protein and VE-cadherin protein than the untransfected cells as analysed by Western blot analysis. (**B**) An increase in cell migration was observed in MCF-7^HER2^ cells when compared with the parental MCF-7 cells. (**C**) The cell migration assay showed that HER2 overexpression resulted in an increased MCF-7 cell migration rate (2.76-fold, *P* = 0.000) when compared with the control cells.

To verify the observations made in human specimens, the human breast cancer cell line MCF-7 was transfected with the pcDNA3-HER2 mammalian expression plasmid (provided by Dr. Atanasio Pandiella, Salamanca University, Salamanca, Spain). HER2-expressing clones were identified by Western blot analysis (Fig. 3A). Using fibronectin-coated Boyden chambers, an increase in cell migration was observed in MCF-7^HER2^ compared with the parental MCF-7 (Fig. 3B). The cell migration assay showed that HER2 overexpression resulted in a 2.76-fold (*P* = 0.000) increase in MCF-7 cell migration rate (Fig. 3C), indicating that HER2 overexpression promotes more aggressive biological behaviour in breast cancer.

**Fig. 4:**
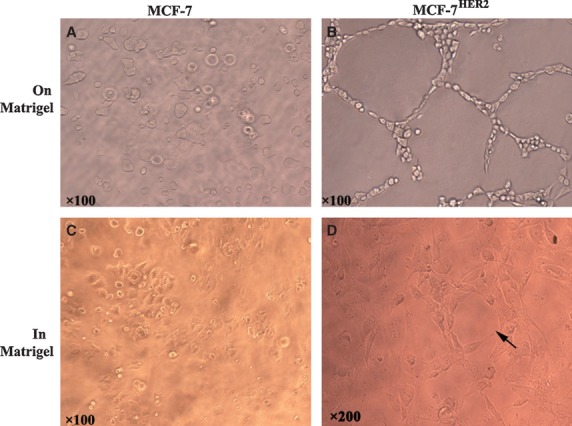
Compared with the parental MCF-7 cells (**A**) and (**C**), MCF-7^HER2^ cells could form vessel-like structures on Matrigel culture in 24 hrs (**B**) and form VM in 3D Matrigel culture in 3–7 days (**D**, black arrow shows vascular tubular channel formation in MCF-7^HER2^ cells).

Notably, MCF-7^HER2^ cells generated patterns consisting of a translucent tubular network when plated on Matrigel, whereas the parental MCF-7 cells did not have the ability to form vessel-like structures (Fig. 4A). MCF-7^HER2^ cells began to form tubular structures in less than 17 hrs when plated on Matrigel, and they formed a characteristic microvascular structure by 24 hrs (Fig. 4B).

Our recent findings, as well as those of other researchers [10, 11] have demonstrated the unusual ability of aggressive cancer cells to form VM in 3D Matrigel cultures. The generation of these channels by epithelial tumour cells in 3D culture could be identified as VM because the 3D culture can better mimic the *in vivo* tumour microenvironment than the two-dimensional culture. In 3D Matrigel culture, MCF-7^HER2^ cells showed stronger VM ability. Vascular tubular channels were formed and evolved dynamically over 3–7 days (Fig. 4D). By contrast, MCF-7 cells, which have very low expression of HER2, did not form tubular networks in the 3D Matrigel for up to 7 days after plating (Fig. 4C). The lumen diameter in the tubular networks of VM widely varied compared with the endothelial cell cords *in vitro*, which typically demonstrate a uniform diameter on most matrices. Our results provide further support on the important role of HER2 in promoting VM formation.

### VE cadherin was up-regulated in MCF-7^HER2^

Vascular endothelial cadherin is a transmembrane glycoprotein expressed in the adherens junctions between vascular endothelial cells. In addition, the VE cadherin exclusively expressed by highly aggressive melanoma cells is critical in melanoma with VM [21, 22]. The VM formation of MCF-7^HER2^ cells in Matrigel suggests that they may have the capacity to undergo differentiation into endothelial cells. To conclusively show that MCF-7^HER2^ cells can acquire an endothelial cell phenotype in the 3D Matrigel culture, analysis of VE-cadherin expression in MCF-7^HER2^ and parental MCF-7 cells was conducted using Western blot. Remarkably, VE cadherin showed a much higher expression in MCF-7^HER2^ cells than in MCF-7 cells (Fig. 3), suggesting that an epithelial-to-endothelial transition occurred in the MCF-7 cells with exogenous HER2 overexpression. Therefore, we conclude that HER2 can regulate VE cadherin, thus promoting VM formation.

## Discussion

The VM phenomena have been reported to be correlated with poor prognosis in patients [23–25]. Many studies have contributed new insights into the underlying molecular pathways supporting VM [13]. Our laboratory has been focusing on understanding the molecular mechanisms that regulate this process [10–12, 14, 15, 23].

In this study, we detected VM formation in invasive breast carcinoma specimens. We found that VM formation correlated closely with clinicopathological parameters (lymph node-positive and advanced stage), which have been traditionally associated with poor prognosis. Notably, we found that the VM-positive rate increased with an increase in HER2 expression. Similarly, the median number of VM channels was greatest in the HER2 3+ tumours, indicating that HER2 contributes to VM formation in breast cancer. HER2 overexpression is associated with adverse outcomes, and their presence in tumours is associated with a shortened disease-free interval and poor survival in patients [26–28]. Therefore, the greater number of VM identified in primary human invasive breast cancer specimens that expressed high levels of HER2 suggest that these VM channels may serve as an alternative means of generating microcirculation, thus facilitating metastasis and inducing adverse outcomes for HER2-positive tumours.

Less invasive MCF-7 cells cannot form VM *in vitro*. With exogenous HER2 introduction into MCF-7 cells, MCF-7^HER2^ cells showed more aggressive biological behaviour, as detected by cell migration assays. MCF-7^HER2^ cells were able to generate VM, and these VM channels were different from the endothelium-derived angiogenic vessels. Furthermore, the MCF-7^HER2^ cells that formed the VM networks expressed VE cadherin, suggesting that HER2 could be a key regulator of VE-cadherin expression and activation. Therefore, we assume that when the growth of endothelium-dependent vessels is insufficient for the rapid proliferation of HER2+ tumour tissues, some tumour cells alter their gene expression programme and the fate of the cells becomes similar to that of the endothelial cells.

We also speculate that HER2 activity regulates invasion, nodal metastasis and the VM potential of invasive breast cancer cells by promoting the expression of VE cadherin. The relationship between HER2 overexpression and VM may be a key factor for HER2+ tumour relapse and progression. Understanding key molecular events may provide additional therapeutic intervention strategies for HER2+ breast cancer.
